# Supplementary material for: Force and kinetics of fast and slow muscle myosin determined with a synthetic sarcomere–like nanomachine
Source: Commun Biol. 2024 Mar 23;7:361. doi: 10.1038/s42003-024-06033-8 (PMC10960843; doi:10.1038/s42003-024-06033-8)
Supplement: Supplementary file 2 — Supplementary Information [file 42003_2024_6033_MOESM2_ESM.pdf]

Supplementary Information

Supplementary Table 1

Supplementary Table 1. Energetic cost of isometric contraction in slow and fast mammalian skeletal muscle in terms of rate of ATP hydrolysis per myosin motor, ϕ, and ratio of ϕ of fast over ϕ of slow muscle (R). Data from the literature (ref, in brackets) except the last row that reports the data from the nanomachine simulation

ϕ (s^{-1})	[ref.]	fast	slow	R
Mouse muscle 21 °C	[1]	12.4	2.47	5.0
Mouse muscle 25 °C	[2]	13.3	2.95	4.5
Rat muscle 27 °C	[3]	12.5	2.3	5.4
Rat skinned fibre 12 °C	[4]	1.28	0.25	5.12
Rabbit skinned fibre 12 °C	[5]	1.79	0.23	7.78
Human skinned fibre 12 °C	[6]	3.22	0.65	4.95
Nanomachine 24 °C		6.0	2.3	2.6

Supplementary Note 1

Additional information on the stochastic model

The quantities $T(\boldsymbol{n'}|\boldsymbol{n})$, as introduced in the main body of the paper, stand for the transition probabilities from the state $\boldsymbol{n}$ to the state $\boldsymbol{n'}$. In the following, to identify the arrival/departure state $\boldsymbol{n'}$ we solely highlight the individual component of the vector $\boldsymbol{n}$ that changes due to the considered reaction. The transition rates as stemming from the chemical equations that define the stochastic model under investigation take the following explicit form:

$$
\begin{array}{ll}
\textit{(1) ATTACHMENT} & T(n_1+1|\boldsymbol{n}) = k_1\,\dfrac{n_D}{N} = k_1\Big[1-\dfrac{1}{N}(n_1+n_2)\Big] \\
\textit{(2) DETACHMENT} & T(n_1-1|\boldsymbol{n}) = k_{-1}\,\dfrac{n_1}{N} \\
\textit{(3) CONVERSION} & T(n_1-1,n_2+1|\boldsymbol{n}) = k_2\,\dfrac{n_1}{N} \\
\textit{(4) CONVERSION} & T(n_1+1,n_2-1|\boldsymbol{n}) = k_{-2}\,\dfrac{n_2}{N} \\
\textit{(5) DETACHMENT} & T(n_2-1|\boldsymbol{n}) = k_3\,\dfrac{n_2}{N}
\end{array}
\tag{1}
$$

The governing master equation can be hence written in the following explicit form:

$$
\begin{aligned}
\frac{\partial P(\boldsymbol{n},t)}{\partial t} = \Big[& T(\boldsymbol{n}|n_1-1)P(n_1-1;t) - T(n_1+1|\boldsymbol{n})P(\boldsymbol{n},t)+ \\
& + T(\boldsymbol{n}|n_1+1)P(n_1+1;t) - T(n_1-1|\boldsymbol{n})P(\boldsymbol{n},t)+ \\
& + T(\boldsymbol{n}|n_1+1,n_2-1)P(n_1+1,n_2-1;t)+ \\
& - T(n_1-1,n_2+1|\boldsymbol{n})P(\boldsymbol{n},t)+ \\
& + T(\boldsymbol{n}|n_1-1,n_2+1)P(n_1-1,n_2+1;t)+ \\
& - T(n_1+1,n_2-1|\boldsymbol{n})P(\boldsymbol{n},t)+ \\
& + T(\boldsymbol{n}|n_2+1)P(n_2+1;t) - T(n_2-1|\boldsymbol{n})P(\boldsymbol{n},t)\Big] \ .
\end{aligned}
\tag{2}
$$

Additional information on the adiabatic approximation

As anticipated in the main text, the validity of the adiabatic elimination was postulated even in the absence of a clear time scales separation of the dynamics of the system, and here we test the validity of this approach, for the operating conditions, in reproducing the correct time scale of the dynamics of the concentration for motors in A_2. In the Supplementary Figure 1 it is shown the evolution of the concentration of the motors in the force generating configuration A_2, from a initial condition with all the motors detached from the actin, from which it is possible to estimate the time scale on which the stationary state is approached.

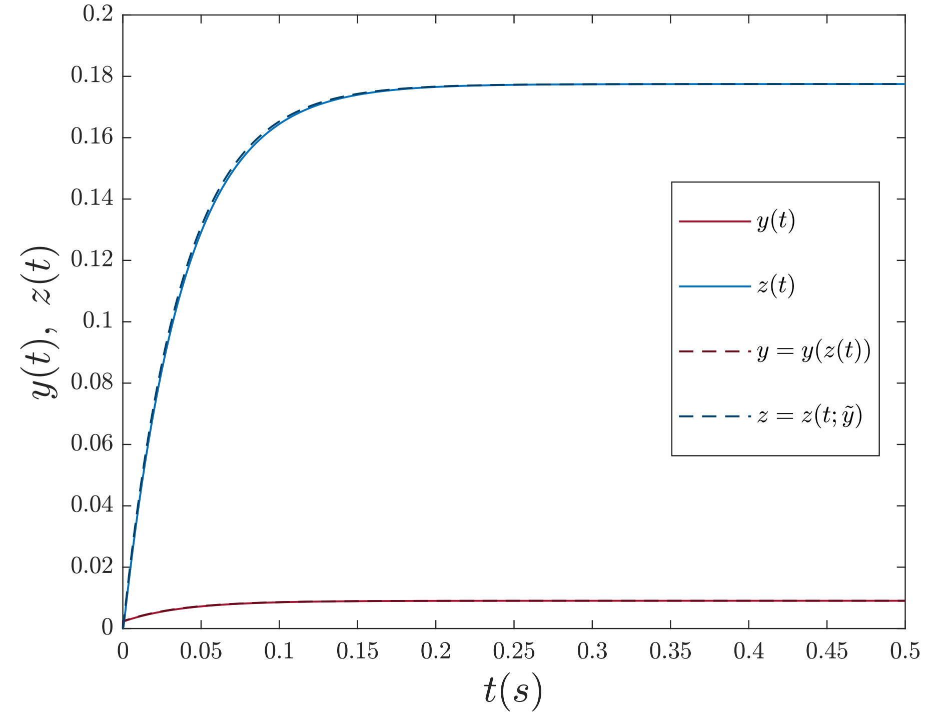

Supplementary Figure 1. ***Checking the adiabatic approximation.***
In red (solid and dashed lines) the evolution of the concentration of motors in configuration A_1, in blue (solid and dashed lines) the evolution of the concentration of motors in configuration A_2. The solid lines represent the integration of the dynamical system without any approximation, the dashed lines correspond to the integration of the dynamical system when the adiabatic approximation is adopted. The approximation allows to reproduce the correct the time scale at which the concentration of motors in A_2 reaches the stationary state.

Additional information on the structure of the stochastic matrix

As remarked in the main body of the paper for each possible value of n_1, n_2 can assume values in the range $[0, N - n_1]$. Hence, the total number of possible states of the system is $M = (N+1)(N+2)/2$. Consider the relevant setting $N = 16$. We thus have to deal with $M = 153$ possible states as listed in the following:

$m \in [1, 153]$	$(n_1,\ n_2)$	
1	$(0, 0)$	
2	$(0, 1)$	$k = 0$
$\vdots$	$\vdots$	
17	$(0, 16)$	
18	$(1, 0)$	
$\vdots$	$\vdots$	$k = 1$
33	$(1, 15)$	
34	$(2, 0)$	
$\vdots$	$\vdots$	$\vdots$
150	$(14, 2)$	
151	$(15, 0)$	$k = 15$
152	$(15, 1)$	
153	$(16, 0)$	$k = 16$

(3)

Focus now on the generic element W_{lm} that enters the definition of matrix $\mathbb{Q}$. Assume in particular m to label the reference initial state; l identifies the state that can be eventually reached through the chemical dynamics. Five possible types of transitions exist, organised in $k = N + 1$ blocks, which corresponds to the selected value of n_1, while n_2 can freely varies within $[0, N - n_1]$:

$$l_1 = m^{max}(k+1) + n_2 + 1$$

$$l_2 = m^{max}(k) - n_2^{max} + n_2 + 1$$

$$l_3 = m^{max}(k) - n_2^{max} + n_2 + 2$$

$$l_4 = m^{max}(k+1) + n_2$$

$$l_5 = m - 1$$

where we denote $m^{max}(k)$ the largest possible index as associated to block k, for the selected choice of m; n_2^{max} stands the largest values that can eventually take the discrete variable n_2. Hence:

$$n_2^{max} = N - n_1$$

$$m^{max}(k+1) = m^{max}(k) + N + 1 - n_1$$

The non trivial elements $W_{m,l}$ are hence:

$$W_{l_1,m} \equiv T(n_1+1, n_2|n_1, n_2) = \frac{k_1}{N}\big(N - n_1 - n_2\big) \qquad \begin{cases} n_2 \in \big[0, n_2^{max}-1\big] \\ n_1 \in \big[0, N-1\big] \end{cases}$$

$$W_{l_2,m} \equiv T(n_1-1, n_2|n_1, n_2) = \frac{k_{-1}}{N} n_1 \qquad \begin{cases} n_2 \in \big[0, n_2^{max}\big] \\ n_1 \in \big[1, N\big] \end{cases}$$

$$W_{l_3,m} \equiv T(n_1-1, n_2+1|n_1, n_2) = \frac{k_2}{N} n_1 \qquad \begin{cases} n_2 \in \big[0, n_2^{max}\big] \\ n_1 \in \big[1, N\big] \end{cases}$$

$$W_{l_4,m} \equiv T(n_1+1, n_2-1|n_1, n_2) = \frac{k_{-2}}{N} n_2 \qquad \begin{cases} n_2 \in \big[1, n_2^{max}\big] \\ n_1 \in \big[0, N-1\big] \end{cases}$$

$$W_{l_5,m} \equiv T(n_1, n_2-1|n_1, n_2) = \frac{k_3}{N} n_2 \qquad \begin{cases} n_2 \in \big[1, n_2^{max}\big] \\ n_1 \in \big[0, N\big] \end{cases}$$

Given the above structure, it is possible to identify for every choice of m, the corresponding combination of n_1 and n_2, and associate the $m-$component of vector $\boldsymbol{P}(t)$ to a specific state (n_1, n_2). That is because the $m^{max}(k)$ are the partial sums of the finite sequence:

$$m^{max}(k) = \sum_{i=0}^{k} N + 1 - i \qquad i \in \{0, \ldots, N\}$$

for $k = 0, \ldots, N$.
For a given m, we thus identify the index k that matches the relation:

$$m^{max}(k) \geq m$$

and then set:

$$\begin{cases} n_1 = k \\ n_2 = m - m^{max}(k-1) - k \end{cases} \tag{4}$$

The stationary solution of the stochastic dynamics is found as the eigenvector of $\mathbb{Q}$ associated to the null eigenvalue.

Deterministic and stochastic simulations of the underlying model

Consider first the set of differential equations that represents the mean field approximation for the examined system. These equations can be numerically integrated, for a representative choice of the chemical reaction parameters, so as to resolve the temporal evolution of the concentrations of the motors, in configurations D, A_1 and A_2. As shown in Supplementary Figure 2, after a transient, the concentration of the motors in the various populations approach their stationary state x^*, y^*, z^*.

We now turn to studying the stochastic model that implements the microscopic dynamics described by the chemical equations (1) in the main text. It is possible to numerically simulate a single stochastic orbit of the considered dynamics (working with the same kinetic constants as assumed in the the mean field simulations) via the celebrated Gillespie algorithm. In doing so one eventually obtains the time evolution of the (discrete) concentration of molecular motors in each configuration, i.e. $n_D(t)/N$, $n_1(t)/N$, $n_2(t)/N$. A typical solution is displayed in Supplementary Figure 3. As expected the stochastic trajectories fluctuate around the corresponding deterministic orbit (solid lines). The observed fluctuations are a material imprint of the inherent discreteness of the simulated system.

The distribution of fluctuations can be numerically accessed from individual stochastic simulation, by averaging over a large set of independent stochastic realisations. In Supplementary Figure 4 the equilibrium distribution of the fluctuations (i.e. the fluctuations displayed around the fixed point, once the initial transient has faded away) is depicted and compared to the analytical solution obtained from the governing master equation, via the procedure discussed above. The agreement is satisfying and testifies on the correctness of the proposed analytical treatment.

We are now in the position to simulate also the temporal series of the force of the ensemble, that is obtained by assigning to each individual motor the force that it is able to exert, based on its configuration (as stipulated by the stochastic dynamics), and following the prescriptions described in the Results. The time series of the force exerted by motors of the populations A_1 and A_2 is displayed in Supplementary Figure 5, for the specific choice of the parameters here operated.

By accessing the temporal evolution of the force, including the equilibrium fluctuations, one can recover key information on the underlying structural and chemical parameters. As shown in the main body of the paper, this corresponding to solving an inverse problem, from the observed force back to the relevant parameters.

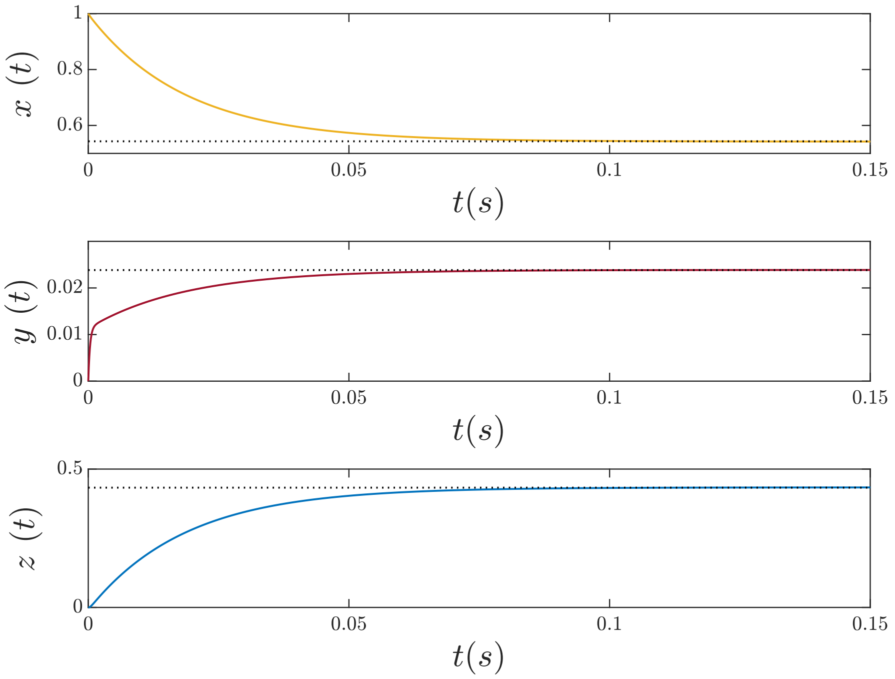

Supplementary Figure 2. ***Temporal behaviour of the mean field concentrations of motors in different states.***
The solid yellow line is the evolution of motors in the detached configuration D (yellow); the solid red and blue lines are the evolution of motors in the configuration A_1 *and* A_2 *respectively. The dotted lines correspond to the stationary state values* x^*, y^*, z^*.

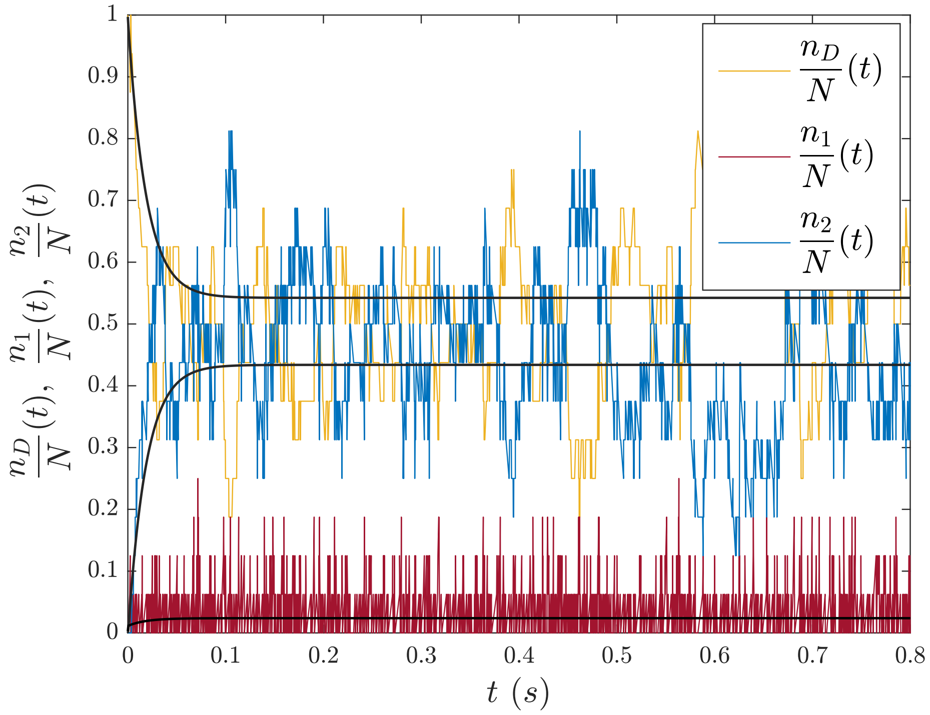

Supplementary Figure 3. ***Stochastic simulations obtained with the Gillespie algorithm.*** *Temporal behaviour of the concentration of the three populations of motors in each configuration: D (yellow line), A1 (red line), A_2 (blue line). The black solid lines represents the deterministic evolution of the concentrations.*

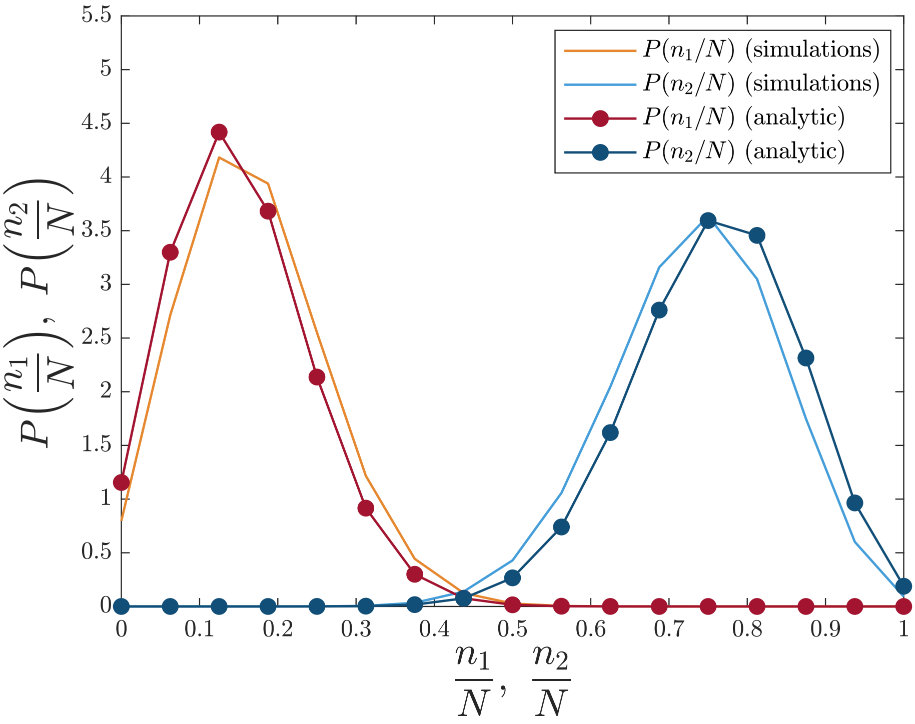

Supplementary Figure 4. ***Checking the theory predictions vs. stochastic simulations.*** *Comparison between the probability distributions of the concentrations of motors in the force–generating configurations as obtained from the simulated dynamics and the stationary solution of the master equation. Normalised histograms (light coloured lines) refers to the populations of motors in configurations* A_1 *(in red) and* A_2 *(in blue), as obtained from the simulated dynamics for a suitable choice of the kinetic parameters. Dark coloured lines (with symbols) stand for the homologous distribution as derived from the stationary solution of the master equation.*

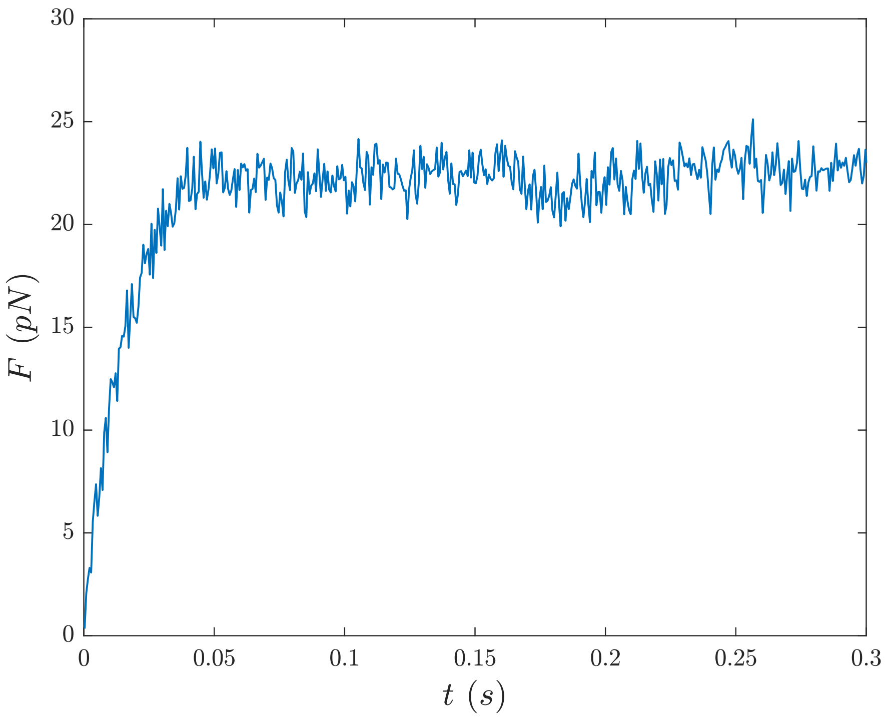

Supplementary Figure 5. ***Force development as obtained by averages of stochastic simulations.***
The trajectory has been obtained averaging over 100 *simulations. The force is measured in pN and it is exerted by a collection of* $N = 16$ *molecular motors with a suitable choice of the kinetic parameters.*

Analytical characterisation of the force probability distribution

As discussed in the main body of the paper, the fluctuations of the force around the average value stem from finite size corrections. To estimate the distribution we focus on $k \in [1, N]$ force–generating distinct motors and postulate that each of them can exert a uniform, randomly selected force f. For each choice of k, one can compute the distribution of the force $\Pi_k(f)$ exerted by the k motors. This is a particular case of the more general problem of calculating the probability distribution for the total force exerted by all the n_1 and n_2 molecular motors in the force–generating configurations A_1 and A_2. In this section we discuss the problem under this general perspective. To this end we denote by f_1 the random force uniformly distributed in the interval $\mathcal{I}_1$ and exerted by the motors in configuration A_1 and by f_2 the one extracted from the interval $\mathcal{I}_2$, exerted by the motors in the configuration A_2. As suggested in[7] we are dealing with the problem of finding the probability distribution of the sum of n random variables Y_i, for $i = 1, \ldots, n$ each of them uniformly distributed in the interval $[b_i, c_i]$. In our case we have only two classes of variables Y_i: the ones relative to the forces exerted by motors in the configuration A_1, and those relative to the forces exerted by motors in the configuration A_2, so that:

$$\sum_{i=1}^{n} Y_i = \sum_{i=1}^{n_1} Y_i + \sum_{i=n_1+1}^{n} Y_i$$

and:

$$Y_i = \begin{cases} f_1 & \text{and} \quad [b_i, c_i] = [-f_0, f_0] \quad \text{if} \quad i = 1, \ldots, n_1 \\ \\ f_2 & \text{and} \quad [b_i, c_i] = \left[\dfrac{f_0}{10}, f_0\right] \quad \text{if} \quad i = n_1 + 1, \ldots, n \end{cases} \tag{5}$$

We can observe that the probability distributions of the sum of the variables Y_i is the same as the probability distribution of the sum of the variables $X_i = Y_i - b_i$, which are defined in the intervals $[0, a_i]$, where $a_i = c_i - b_i$.
Introduce the sum s:

$$s = \sum_{i=1}^{n} X_i$$

with $n \geq 2$. Then the distribution of the sum as defined above reads[7]:

$$\Pi_n(s; n_1, n_2) = \frac{1}{(n-1)!} \frac{1}{(a_1)^{n_1} (a_2)^{n_2}} \left[s^{n-1} + \sum_{k=1}^{n} (-1)^k \left(\sum_{J_k} \Big(s - \sum_{l=1}^{k} a_{j_l} \Big)_+ \right)^{n-1} \right] \tag{6}$$

where we adopted the notation: $(f)_+ = max\{0, f\}$.
From this expression it is possible to compute the probability distribution for the sum of the variables of our interest:

$$\sum_{i=1}^{n} Y_i = \sum_{i=1}^{n} \Big(X_i + b_i \Big) = \sum_{i=1}^{n} X_i + \sum_{i=1}^{n_1} b_i + \sum_{i=n_1+1}^{n} b_i = s + n_1 b_1 + n_2 b_2$$

where $b_1 = -f_0$ and $b_2 = \frac{f_0}{10}$.
If we consider just one class of variables Y_i, meaning if we consider only the forces exerted by one of the two force–generating populations of motors, the distributions Π_n can be computed as a specific case of the generalisation of the Irwin–Hall distribution,[8], the uniform sum distribution. These refer to the sum of n random variables x_i, each of them defined in the interval $[a, b]$ and take the form:

$$\Pi_n(x) = \frac{1}{b-a}\, g(y; n) \qquad \text{with:} \qquad y = \frac{x - na}{(b-a)} \tag{7}$$

where:

$$g(y, n) = \frac{1}{2(n-1)!} \sum_{k=0}^{n} (-1)^k \binom{n}{k} (y-k)^{n-1} \operatorname{sgn}(y-k) \ .$$

In order to obtain the correct expression for the probability distribution of the total force exerted by both n_1 motors in the configuration A_1 and n_2 motors in the configuration A_2 at a given time, one has to weight the above expressions with the probability to find the system in the considered condition. This latter follows the stationary solution of the master equation as discussed in the main body of the paper. From $\boldsymbol{P}^{st}$, we can in particular extract the probability ρ_k to find $k \leq N$ motors in A_2. This is obtained by summing the elements of $\boldsymbol{P}^{st}$ that refer to a given k, thus accounting for all possible partitioning of the remaining $N-k$ motors among states D and A_1 (see also discussion in the main body of the paper). Labelling these marginal probabilities $(\rho_0, \rho_1, \rho_2, ..., \rho_N)$ one gets:

$$P(F) = \rho_1 \Pi_1(f) + \rho_2 \Pi_2(f) + \cdots + \rho_{16} \Pi_{16}(f) \ = \sum_{i=1}^{N} \rho_i \ \Pi_i(f) \ .$$

where use has been made of the fact that $\Pi_0 = 0$.
The obtained profiles are reported in Supplementary Figure 6 for the relevant case $N = 16$ and for $i = 1, 2, 3, 4$. In Supplementary Figure 7 the global distribution of fluctuations is depicted for a specific choice of the kinetic parameters of the model.

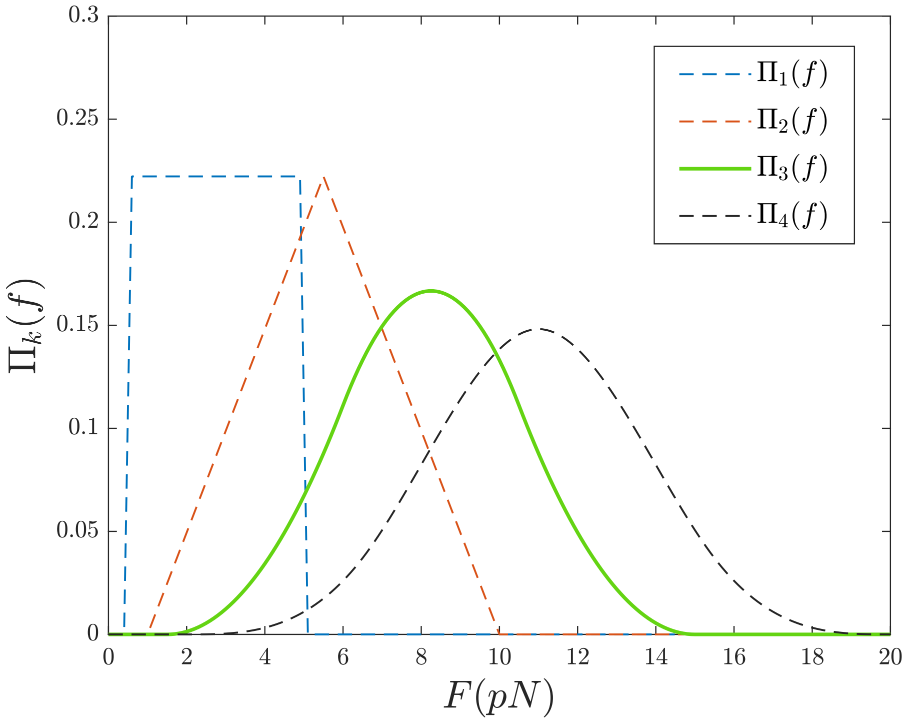

Supplementary Figure 6. ***Many body force distributions.***
The distributions $\Pi_k(f)$ *are plotted for* $k = 1, 2, 3, 4$.

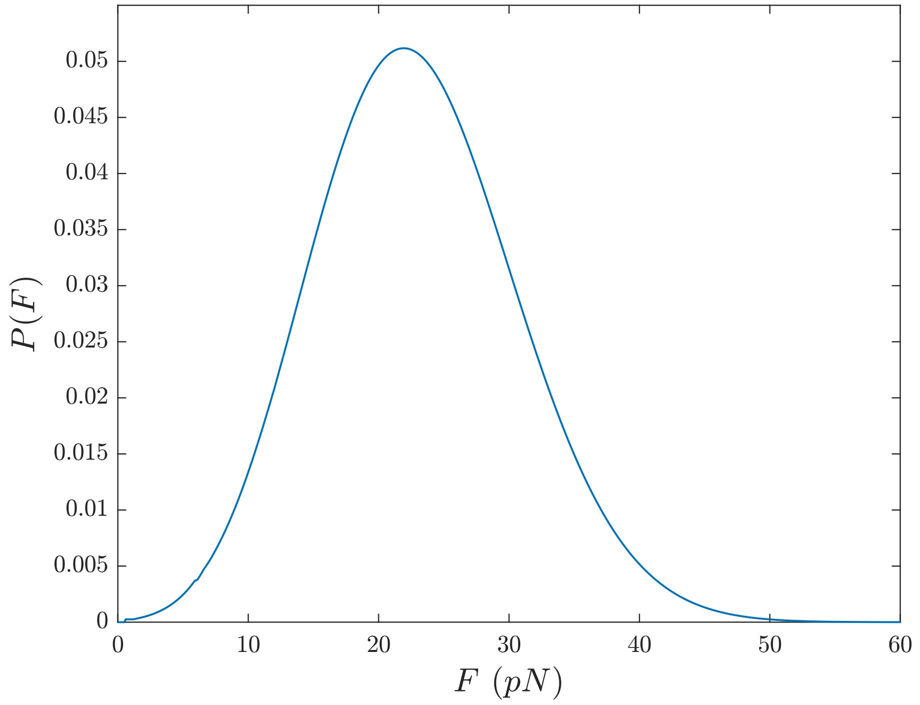

Supplementary Figure 7. ***Theoretical distribution of force fluctuations.***
Probability density function $P(F)$ as resulting from the sum of the $\Pi_k(f)$, for $k = 1, \ldots, N$ weighted with the stationary solution of the master equation for a system of $N = 16$. The adopted parameters are: $f_0 = 6\ pN$, $k_1 = 30\ s^{-1}$, $k_{-1} = 500\ s^{-1}$ $k_2 = 2000\ s^{-1}$ $k_{-2} = 100\ s^{-1}$ $k_3 = 10\ s^{-1}$.

Parameters estimation

The fitting procedure described in Results and applied to the experimental data set of soleus and psoas HMM has been validated against synthetic data, and the details are presented in this Section. The parameters f_0 and r are correctly estimated, as it follows from inspection of Supplementary Table 2, where the error associated with the estimated value of r is the standard deviation. Also the estimated b and a (recomputed from the best fitted values for the kinetic constants) are pretty close to their nominal values as imposed in the simulations, as well as the value of the rate ϕ. Supplementary Figure 8 shows the graphic comparison between the results of the optimisation procedure for the parameters estimation performed under the assumptions of the effective model, and the values of the parameters adopted in the stochastic simulations of the dynamics (these latter are referred to as true parameters, as they are imposed in the simulation and thus known with infinite precision). The symbols refers to the mean and SD of the duty ratio r, the force of a single motor f_0, the rate ϕ and the two parameters a and b that describe the dynamics of the system in the deterministic limit. The blue line represents the value of the corresponding parameter adopted in the simulations.

Supplementary Figure 9 refers to the parameters estimation in the plane (f_0, r), where the solutions resulting from the analysis of the force of the ensemble in the deterministic framework are represented by the solid line, while the symbols shows the value of the parameter f_0 and r that can be estimated by taking into account the fluctuations of the force of the ensemble.

The result of the analysis for different system size N are reported in Supplementary Figure 10: the solid line represent f_0 as a function of N, while the symbols refers to the best fit value of f_0 as determined for different choices of N. We remark that the fitting procedure converges (with the requested limit of precision) only over a finite range of values of N, centred around the value adopted when performing the simulations. This observation implies in turn that we are in a position to obtain a reasonable estimate for the interval of pertinence of N, as follows the procedure outlined above.

Supplementary Table 2. Estimated parameters via the inverse scheme fed with simulated data. Errors are below 10^{-3} if not explicitly provided.

	F_0 (pN)	f_0 (pN)	r	a (s^{-1})	b (s^{-1})	$\phi(s^{-1})$
True parameters	22.9	6.0	0.46	54.7	23.7	13.43
Estimated parameters	22.7	6.1	0.46 ± 0.03	54.8	23.5	13.42

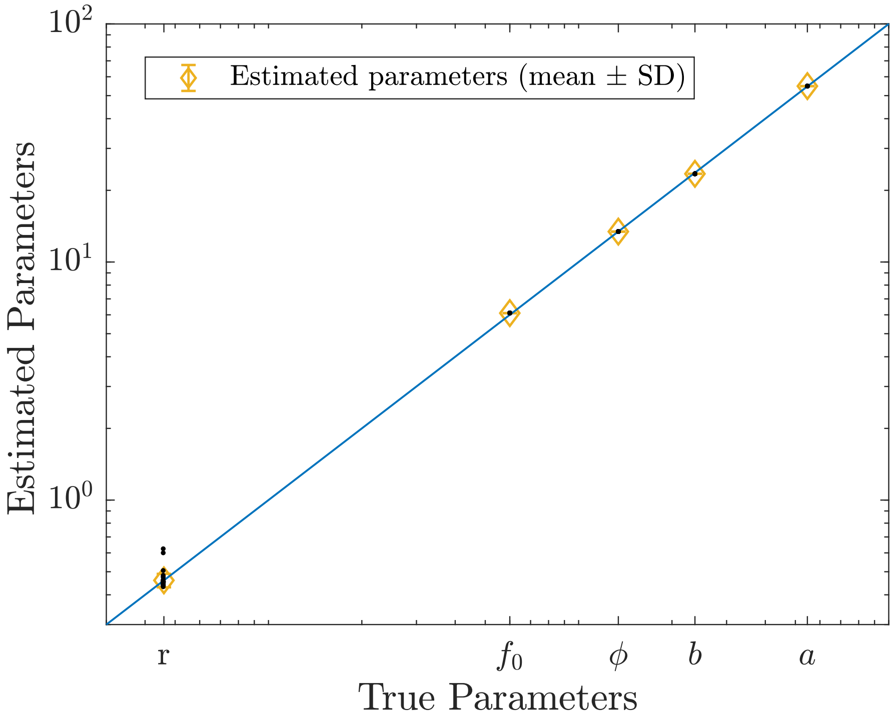

Supplementary Figure 8. ***Results of the optimisation procedure for the parameters estimation.***

The parameters estimation has been performed in the framework of the effective model and the results are identified by the yellow symbols (mean ± SD). Mean and SD for each parameter are computed from 58 *independent iterations of the fitting procedure (black dots). The estimated values are compared with the parameters adopted in the stochastic simulations of the dynamics (blue solid line).*

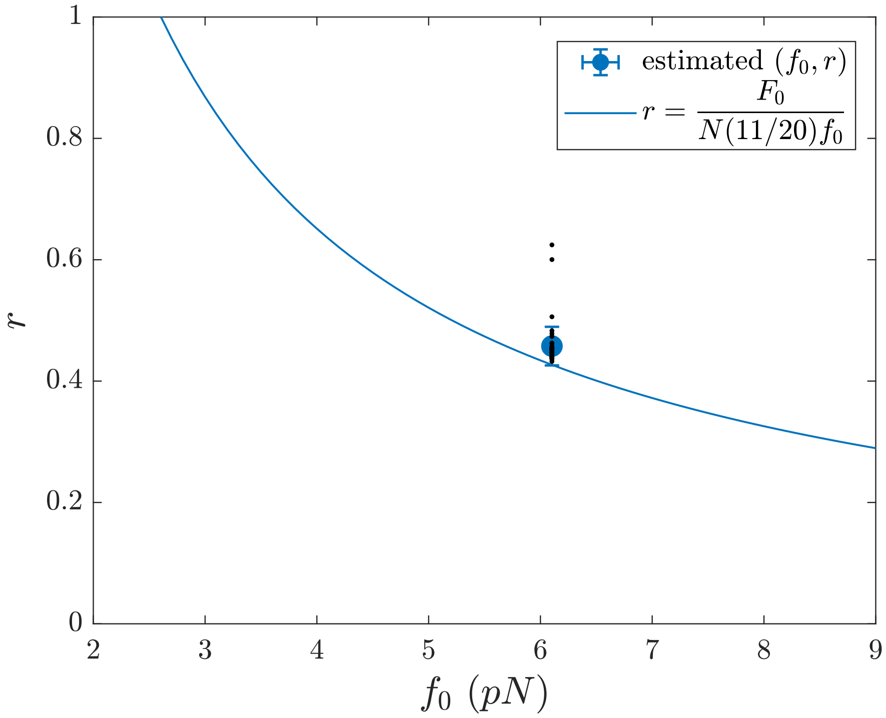

Supplementary Figure 9. ***Parameters estimates in the reference plane*** (f_0, r)***.***
The symbol follows from the integrated fitting strategy that accounts for fluctuations. Mean and SD is computed from 58 *independent iterations of the fitting procedure (black dots). The solid line is the hyperbole populated with the degenerate mean field, hence deterministic solutions. Remark that the fitted symbol is close but not on top of the hyperbole. The observed deviation is eventually due to the residual population* y^* *that is adequately estimated via the generalised fitting strategy base on the stochastic description. The error is obtained from different replica of the stochastic optimisation algorithm.*

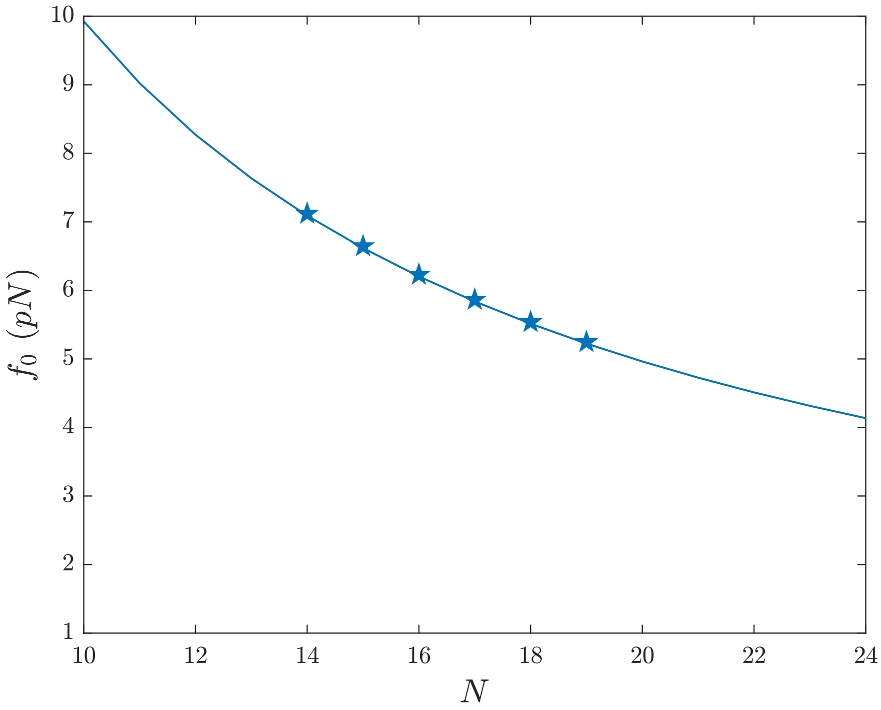

Supplementary Figure 10. ***Checking*** f_0 ***against*** N**.**
The parameter f_0 *is estimated as a function of* N*, applying the inverse scheme to the simulated data.*

Parameters estimation for soleus and psoas HMM data

A representative example of the fitting outcome for the soleus HMM ensemble is reported in the Supplementary Figure 11. The histogram is generated from the experimental data series of the isometric force in the stationary state (i.e. at the isometric plateau). The results of the fitting procedure and the parameters estimation performed on the experimental data sets from rabbit psoas and rabbit soleus are listed in the Supplementary Table 3 and Supplementary Table 4 respectively. The result of the analysis for different system size N are reported in Supplementary Figure 10: the symbols refers to the best fit value of f_0 as determined for different choices of N. The shaded region identifies the portion of the parameters plane where the solutions are expected to be found. Specifically, it is assumed to lay in between the two curves:

$$f_0 N = \frac{20}{11} \langle F_0 \pm 2\Delta F_0 \rangle \left\langle \frac{a}{b} \right\rangle \tag{8}$$

Here the relative error associated with the average value of the quantity $\langle a/b \rangle$ is assumed negligible, as compared to that stemming from the average stationary force, ΔF_0. The histogram computed from the collection of fitted parameters (each choice of symbols refers to a different experimental series) can be conceptualised as an indirect imprint of the degree of experimental variability as associated to f_0 and N.

Supplementary Table 3. Estimated parameters for psoas data

Exp	F_0 (pN)	f_0 (pN)	r	$\phi(s^{-1})$
PSO 1	16.4	5.6	0.36	6.4
PSO 2	16.1	5.8	0.34	6.1
PSO 3	20.7	7.6	0.32	6.2
PSO 4	17.7	6.9	0.32	5.9
PSO 5	19.7	8.0	0.30	5.8
PSO 6	17.0	7.0	0.30	5.8
$mean \pm SD$	17.9 ± 1.9	6.8 ± 1.0	0.32 ± 0.02	6.0 ± 0.2

Supplementary Table 4. Estimated parameters for soleus data

Exp	F_0 (pN)	f_0 (pN)	r	$\phi(s^{-1})$
SOL 1	8.7	1.8	0.55	2.25
SOL 2	10.9	2.5	0.51	2.27
SOL 3	9.1	2.2	0.49	2.29
SOL 4	13.4	3.2	0.49	2.26
SOL 5	9.7	2.3	0.48	2.34
SOL 6	9.3	2.4	0.48	2.23
$mean \pm SD$	10.2 ± 1.7	2.4 ± 0.4	0.50 ± 0.03	2.27 ± 0.04

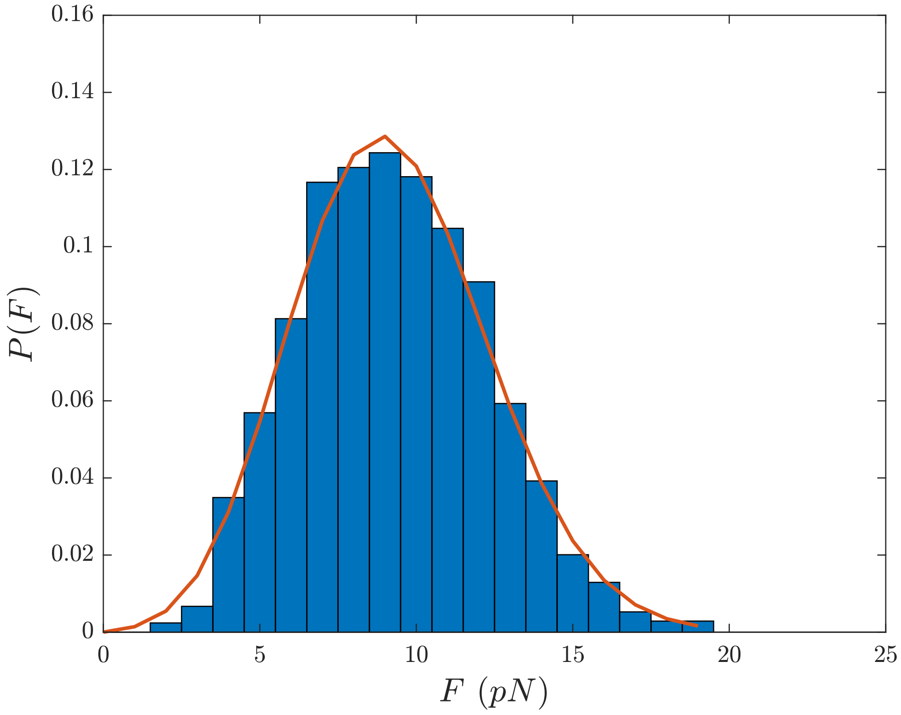

Supplementary Figure 11. ***Result of the fitting procedure on the force of the soleus HMMs ensemble.***
The histogram of the soleus force at the isometric plateau is fitted against the analytical profile, via a self–consistent optimisation procedure which aims at estimating the unknown kinetic parameters.

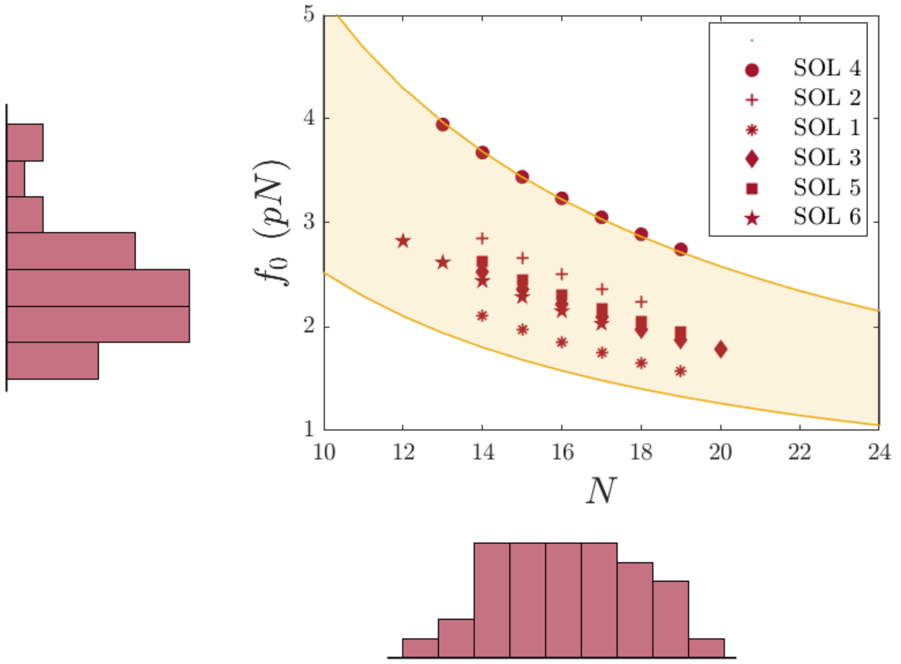

Supplementary Figure 12. f_0 ***vs.*** N ***on the force of the soleus HMMs ensemble.*** *The estimated parameter* f_0 *of the soleus HMMs is plotted as a function of the imposed* N*; each choice of symbols refers to a different experimental series. The shaded region is drawn from the theoretical curve that resolve the dependence of* f_0 *on* N*.*

Supplementary Note 2

Rate of force development *in situ*

The time course of isometric force redevelopment following a release that drops the isometric force to 0 was determined in Ca^{2+}–activated demembranated fibres from the same slow and fast muscles of the rabbit used to extract the myosin isoforms for the nanomachine.
As previously described[9–11], small bundles dissected from the two muscles were stored in skinning solution containing 50% glycerol at −20 °C for 3–4 weeks and single fibres were prepared just before the experiment. A fibre segment 4–6 mm long was clamped at its extremities by T–clips and mounted between the lever arms of a loudspeaker motor and a capacitance force transducer[12]. To prevent sliding of the ends of the fibre segment inside the clips and minimise the shortening of the activated fibre against the damaged sarcomeres at the ends of the segment during force development, the extremities of the fibre were fixed first with a rigor solution containing glutaraldehyde and then glued to the clips with shellac dissolved in ethanol. Fibres were activated by temperature jump using a solution exchange system as previously described[9]. A striation follower[13] allowed nanometre–microsecond resolution recording of length changes in a selected population of sarcomeres.
The composition of the solutions has been reported previously ([11], Supplementary Table 1, 25 °C). The increase of interfilamentary distance following cell membrane permeabilisation was reversed by the addition of the osmotic agent Dextran T–500 (4% weight/volume).
The rate of force development was determined on the isometric force rise recorded after superimposing on the isometric contraction of the maximally Ca^{2+}–activated fibre (pCa 4.5) a fast ramp shortening (5–6% of the initial fibre length) able to drop the force to zero (Supplementary Figure 13).

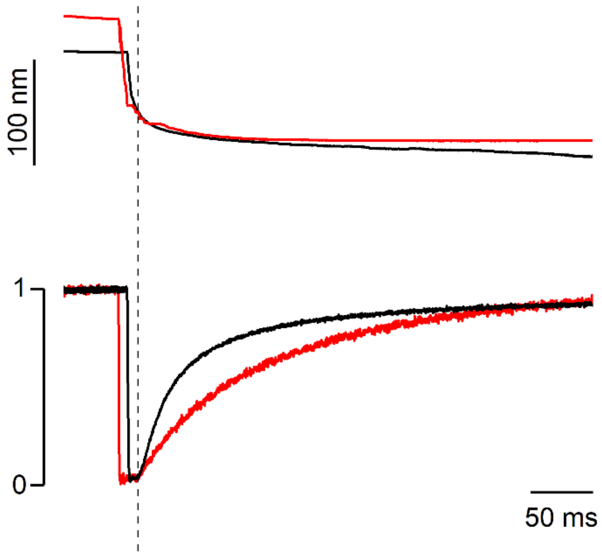

Supplementary Figure 13. ***Time course of force redevelopment in fast and slow skinned fibres.***
Force redevelopment (lower traces) and corresponding half–sarcomere shortening (upper traces) after a period of unloaded shortening in a skinned fibre from rabbit psoas (black traces) and soleus (red traces) muscles. The vertical line indicates the time at which the force development starts. Force is normalised for the isometric value (T_0) before the imposed large shortening (~ 5% of the fibre length or ~ 60 nm per hs). Further shortening against end compliance during force redevelopment was 29 ± 6 nm per hs ($n = 4$) and 19 ± 3 nm per hs ($n = 11$) in fast and slow fibres respectively. T_0 was 276 ± 44 kPa and 195 ± 26 kPa in fibres from psoas and soleus respectively. Temperature, 25.2 °C. 4% dextran T–500 was added to reduce the lateral filament spacing of the relaxed fibre to the value before skinning.

References

1. Barclay, C., Constable, J. & Gibbs, C. Energetics of fast-and slow-twitch muscles of the mouse. *The Journal of physiology* **472,** 61–80 (1993).
2. Barclay, C., Woledge, R. & Curtin, N. Is the efficiency of mammalian (mouse) skeletal muscle temperature dependent? *The Journal of physiology* **588,** 3819–3831 (2010).
3. Wendt, I. & Gibbs, C. Energy production of mammalian fast-and slow-twitch muscles during development. *American Journal of Physiology-Legacy Content* **226,** 642–647 (1974).
4. Bottinelli, R, Betto, R, Schiaffino, S & Reggiani, C. Unloaded shortening velocity and myosin heavy chain and alkali light chain isoform composition in rat skeletal muscle fibres. *The Journal of physiology* **478,** 341–349 (1994).
5. Reggiani, C., Bottinelli, R. & Stienen, G. J. Sarcomeric myosin isoforms: fine tuning of a molecular motor. *Physiology* **15,** 26–33 (2000).
6. He, Z.-H., Bottinelli, R., Pellegrino, M. A., Ferenczi, M. A. & Reggiani, C. ATP consumption and efficiency of human single muscle fibers with different myosin isoform composition. *Biophysical journal* **79,** 945–961 (2000).
7. Sadooghi-Alvandi, S., Nematollahi, A., Habibi, R, *et al.* On the distribution of the sum of independent uniform random variables. *Statistical papers* **50,** 171–175 (2009).
8. Hall, P. The distribution of means for samples of size n drawn from a population in which the variate takes values between 0 and 1, all such values being equally probable. *Biometrika,* 240–245 (1927).
9. Linari, M., Caremani, M., Piperio, C., Brandt, P. & Lombardi, V. Stiffness and fraction of myosin motors responsible for active force in permeabilized muscle fibers from rabbit psoas. *Biophysical journal* **92,** 2476–2490 (2007).
10. Percario, V. *et al.* Mechanical parameters of the molecular motor myosin II determined in permeabilised fibres from slow and fast skeletal muscles of the rabbit. *The Journal of Physiology* **596,** 1243–1257 (2018).
11. Caremani, M. *et al.* The force of the myosin motor sets cooperativity in thin filament activation of skeletal muscles. *Communications Biology* **5,** 1266 (2022).
12. Lombardi, V. & Piazzesi, G. The contractile response during steady lengthening of stimulated frog muscle fibres. *The Journal of physiology* **431,** 141–171 (1990).
13. Huxley, H. *et al.* Millisecond time-resolved changes in x-ray reflections from contracting muscle during rapid mechanical transients, recorded using synchrotron radiation. *Proceedings of the National Academy of Sciences* **78,** 2297–2301 (1981).
